# Information Phage Therapy Research Should Report

**DOI:** 10.3390/ph10020043

**Published:** 2017-04-30

**Authors:** Stephen T. Abedon

**Affiliations:** Department of Microbiology, The Ohio State University, Mansfield, OH 44906, USA; abedon.1@osu.edu; Tel.: +1-419-755-4343

**Keywords:** bacteriophage therapy, biocontrol, biological control, peer review, phage therapy, pharmacology, preclinical development, publication, regulation

## Abstract

Bacteriophages, or phages, are viruses which infect bacteria. A large subset of phages infect bactericidally and, consequently, for nearly one hundred years have been employed as antibacterial agents both within and outside of medicine. Clinically these applications are described as phage or bacteriophage therapy. Alternatively, and especially in the treatment of environments, this practice instead may be described as a phage-mediated biocontrol of bacteria. Though the history of phage therapy has involved substantial clinical experimentation, current standards along with drug regulations have placed a premium on preclinical approaches, i.e., animal experiments. As such, it is important for preclinical experiments not only to be held to high standards but also to be reported in a manner which improves translation to clinical utility. Here I address this latter issue, that of optimization of reporting of preclinical as well as clinical experiments. I do this by providing a list of pertinent information and data which, in my opinion, phage therapy experiments ought to present in publications, along with tips for best practices. The goal is to improve the ability of readers to gain relevant information from reports on phage therapy research, to allow other researchers greater potential to repeat or extend findings, to ease transitions from preclinical to clinical development, and otherwise simply to improve phage therapy experiments. Targeted are not just authors but also reviewers, other critical readers, writers of commentaries, and, perhaps, formulators of guidelines or policy. Though emphasizing therapy, many points are applicable to phage-mediated biocontrol of bacteria more generally.

## 1. Introduction

Preclinical reports on phage therapy [1,2] or, more broadly, on phage-mediated biocontrol of bacteria [3], often place much emphasis on phage isolation as well as phage molecular characterization, along with phage organismal properties including, in particular, phage host range [4,5]. These emphases are fine and appropriate as it is important to identify candidate phage isolates, ideally in relatively large numbers, which are capable of getting a designated antibacterial job done [6]. Phages thus should have adequate antibacterial performance characteristics and also should be lacking in harmful aspects, such as toxicities towards animals.

Notwithstanding these preliminaries, it is important as well to effectively perform and describe actual, in situ antibacterial experiments. I thereby provide, here, a list of items which I hope collectively may serve as a useful framework for researchers reporting on the results of phage therapy experiments. These I discuss under the headings Phages, Host range, Formulated products, Titers, Subjects, Bacteria, Dosing, Treatments, Pharmacology, and Efficacy, as well as Discussions. Consideration of these various issues I derive in part from Abedon [7,8,9,10] as well as Payne and Jansen [11,12], Mirzaei and Nilsson [5], Brown-Jaque et al. [13], and also Adriaenssens and Brister [14]. Many of the points made should be obvious, some perhaps less so.

For the sake of clarification, note that dosing is of formulated products, which consist of more than just phage virions, and treatments can consist of more than simply phage dosing. Bacteria include those used for phage characterization, phage propagation, bacterial challenge, or which are naturally present in individuals or environments, but in any case are referred to as hosts. The “hosts” for bacteria, that is, to-be-treated individuals, instead are referred to as treatment subjects. Furthermore, an infection is *by* bacteria rather than phage infection *of* bacteria. Emphasis is placed on improving experiment reproducibility, but validity less so, that is, towards increasing experiment repeatability and subsequent extension rather than critically addressing experiment utility [15].

Ultimately the point of preclinical as well as phases I and II clinical trials is to provide justification—in terms of both safety and efficacy—for further preclinical and clinical trials, and their appropriate design and reporting are crucial to general phage therapy success. Consistent inclusion of relevant details within phage therapy publications, or simply being aware of their importance, should allow for improved reader comprehension, more consistent experiment replication, and easier translation of phages and protocols to real-world applications. Not all details are necessarily applicable to all studies, levels of rigor appropriately should increase in the course of development of products or procedures, and case studies of, for example, compassionate care use of phage therapy clinically will justifiably possess different reporting goals from those of commercial or public sector research and development. Nonetheless, the hope is that researchers, relatively early during studies rather than solely at the point of manuscript preparation, will both consider and benefit from the various issues as discussed.

## 2. Considerations

### 2.1. Phages

Studies should describe both what specific phage isolates were used and why they were used. Points to address are discussed as follows.

#### 2.1.1. Source

Where did the phage or phages used come from? This can include where geographically as well as from what types of environments they were isolated as well as when the isolation took place. Especially important is who they were obtained from if from other phage collections. In part these latter details can be relevant because phages can evolve, resulting in variation among strains obtained from different laboratories as well as, without proper precaution, variation within laboratories. Useful as well, and especially for less well-studied phages, is a reasonably comprehensive listing of publications in which study of the phages used is reported, including publications documenting original isolation. The latter not only can be helpful to readers but also helps to address the problem that in numerous cases phage names are not unique to a particular isolate. Such documentation therefore can be useful towards keeping track of a phage’s pedigree. Presumably, that is, researchers working with a given phage will have greater knowledge of that phage, its origin, and who else has worked with it than will the general reader. That knowledge should be shared. For help in naming new phages, see [16].

#### 2.1.2. Characterization

How were phages characterized in vitro? This includes determinations of phage “organismal” characteristics such as burst size, latent period, and adsorption rates along with the conditions in which these characteristics were determined, including, as possible, the similarity of those conditions, and hosts, to those expected during treatment in situ. The details of these characterizations can be important especially since different approaches can yield different types of information, e.g., as considered below, spot testing versus non-spot-based approaches to phage host range determination. If such characterization procedures have been repeated from as previously published, then comparisons to preceding results could be included as well. Employing not only the same procedures but also the same phages as used by others, in parallel with characterizing one’s own phages, can also serve as a means of validating experimental efforts, that is, by demonstrating conformity with previously published results. Authors generally should strive to include not just what has been done for that study but also what others have equivalently reported on the characteristics of a given phage.

#### 2.1.3. Genomics

How were phages characterized bioinformatically? For bioinformatic characterization, the key issues will be to avoid phages possessing genes which are thought or known to bestow undesirable characteristics, such as the encoding of bacterial virulence factors. As part of this process, genes for which a function could not be assigned should be indicated, including quantitatively (i.e., the number of such genes), since in these cases undesirable characteristics cannot be excluded bioinformatically. In principle, at least, it may be possible as well to identify genetic similarities with previously characterized phages, particularly phages for which earlier therapeutic experience may or may not have been favorable. For phages possessing completed sequences, what is the GenBank accession number, or alternative for sequences deposited in the DNA Database of Japan (DDBJ) or the European Nucleotide Archive (ENA)? The publication of bioinformatic information as separate from actual phage therapy studies—or indeed publication of non-therapeutic phage characterization in general—is to be encouraged and especially so to the extent that this allows for more comprehensive reporting on that information. This can include taking into account not just what has been done or found within a given study but also overviews, along with comparisons to related information, as published by others on the same or similar phages.

#### 2.1.4. Justification

By what criteria, inclusion as well as exclusion, were employed phages chosen rather than alternatives? Were they chosen from a larger group of potential therapeutic phages, or instead were experiments undertaken for the sake of repeating or extending previous efforts using the same phages? If not the latter, then did these phages consist of ones which had the most appropriate properties—indicating in detail what properties those are—or simply were all of the phages that were obtained employed in experiments? At a minimum, it is useful for authors to think about these issues, ideally in some depth, of why one phage was chosen over another. It is also important for the field to build up a published record of such thinking as, ultimately, the strength of phage therapy is based on the potential for researchers to relatively easily identify promising candidate phages. Collective clarification of just what criteria might define promising phages, especially characteristics which can be determined prior to the initiation of animal experiments, should therefore be helpful towards progression of the field.

#### 2.1.5. Stocks

How were phage stocks prepared, that is, how were phages grown in vitro? Include what host was used for preparation, why this host was chosen, and whether the preparation host differed, e.g., from as used in previously published studies using the same phage strains. Of relevance is the potential for host strains to encode or produce virulence factors as well as host possession of restriction modification systems (which can alter phage host ranges) or prophages (which if induced can contaminate stocks). Note that testing for the presence of spontaneously produced temperate phages may be done using a variety of approaches, as can be accomplished both prior to and following growth of intended phage stocks. The presence of prophages can be detected in the course of host sequencing, though existence of prophages is a slightly different issue from that of the presence of induced prophages or the presence of recombined prophage DNA within amplified phage stocks.

### 2.2. Host Range

Host range can be viewed, in pharmacological terms, as equivalent to an antibacterial drug’s spectrum of activity. Indeed, combinations of phages, as cocktails, can possess a collective host range which can be viewed as a *cocktail’s* spectrum of activity, that is, that spectrum of hosts which is impacted by treatment phages. Minimally, tested hosts should include not just bacterial strains used for isolation and amplification but also likely target strains along with similar but non-target species likely to be found in treated environments.

#### 2.2.1. Determination

For individual phages used in phage therapy experiments, what were the determined host ranges and, of equivalent importance, how were those host ranges determined? Authors should be aware in particular that various approaches exist for host range determinations and also that these approaches should be other than solely the use of spot testing (as described below). More generally, keep in mind that different approaches to host range determination can indicate not only different aspects of phage host range, but will tend to do so with different levels of rigor. It can also be helpful, as controls, to test phage stocks in the course of host range testing for contamination with other phage strains, especially other phages as may also be commonly grown in the same laboratory. One in addition can test, in parallel, additional phages with known host ranges, that is, for the sake of comparison with newly characterized phages, towards validating host-range determination efforts, and also to extend the previous characterizations of these additional phages.

#### 2.2.2. Spot Testing

Spot testing, that is, the dropping of small volumes of high numbers of phages onto immature bacterial lawns so as to test for ability to create zones of bacterial inhibition, are excellent for the initial screening of phage host range. The problem with spot testing, however, is particularly one of false positives. True positives should retain relatively strong clearing even upon phage stock dilution and ideally should continue to do so given further dilution to the point of appearance of isolated plaques. Phage productive-infection false positives, by contrast, should simply fade with increasing dilution. Host range determinations using spot testing as a sole indicator, particularly without testing phage stock dilutions for an ability to form plaques, consequently at best represent only preliminary means of phage host range characterization. Spot testing therefore may be permissible for initial analyses, but as phages progress through development, a reliance solely on spot testing as a means of host-range determination should be viewed as increasingly inadequate. In addition, if only spot testing without dilution is used for phage host range determination, then that needs to be explicitly pointed out to the reader along with, ideally, a discussion of the limitations associated with reliance on this technique. It should not, in other words, ever be assumed that spot testing is synonymous with adequate, thorough, or indeed a *default* means of phage host range testing.

#### 2.2.3. Tested Hosts

In host range testing it is important to discuss the diversity of the panel of hosts used. Low tested-host diversity reduces the utility of employing many hosts. Clinically or what otherwise are in situ relevant hosts, tested under in situ pertinent conditions, will also typically be more appropriate for host range testing for phage therapy than hosts or conditions chosen instead merely for convenience. In addition, it can be important to test multiple, ideally diverse strains of a given species, e.g., 3 to 5 of each, rather than just a single representative strain. A single strain within a given species, that is, can easily not be representative of a species as a whole in terms of ability to support phage infection. It also can be helpful to indicate the logic or bases behind the choice of hosts tested. In short, host range testing can only be as good as the appropriateness of the hosts, conditions, and assays used.

#### 2.2.4. Cocktails

A cocktail is a formulated product consisting of a mixture of multiple phages. Cocktails have the utility, especially, of broadening the collective host range of phage formulated products. If cocktails were used in a study, then it is relevant to indicate why cocktails were used rather than so-called monophages. For example, to reduce resistance development in treated bacteria, to allow treatment of a broader range of target bacterial strains, to allow for presumptive treatment of bacterial infections, or indeed all three? To what extent especially do the host ranges of the phages making up a cocktail overlap, and to what degree does the cocktail extend the overall spectrum of activity—number of susceptible bacterial types—of the formulated product in comparison with the individual phage components? More generally, how were the phages chosen for the cocktail, or instead excluded from it? Crucial to include in descriptions of cocktails are the number of phage types present along with relevant per-phage information (including titer) and, if possible, the comparative efficacy performance of the individual phage components of the cocktail. Furthermore, worth mentioning is whether the individual phages were stored separately or instead as a mixture, and if separately then whether the length of storage differed between the different phages. Any additional relevant details concerning cocktail development or properties should, of course, also be provided.

### 2.3. Formulated Products

These, in addition to phages, are materials within which phages are suspended. It is important to unambiguously describe the steps, both methodological and conceptual, in going from phage lysates or purified phage stocks to the formulated products that are applied as antibacterial agents.

#### 2.3.1. Purification

Generally, a requirement for substantial purification of phages as pharmaceutical products, especially for human use, is assumed. Specific methods of purification should be documented in detail. In addition, it can be helpful to indicate what degree of phage losses were observed at separate purification steps. Alternatively, for a number of applications, lower levels of phage purification may be appropriate. In these cases, it remains important to indicate whether purification was performed, and if so, again, then by what means (e.g., just clarification and/or filtration), along with what levels of phage losses resulted. Some explanation of why additional purification was or was not undertaken can be helpful, as well as any limitations that lower levels of purification may place on potential alternative uses of phage products.

#### 2.3.2. Purity

What were the goals of purification if performed? Were those goals met? For example, if the goal of purification was to remove lipopolysaccharide (LPS) from lysates of Gram-negative bacteria, then what LPS concentration remained following purification, what was the efficiency of its removal, was the degree of removal adequate from a clinical perspective, and how were LPS concentrations in these preparations measured? Note that it is possible to do especially first-approximation toxicity testing to determine whether levels of purity are appropriate for intended tasks, e.g., formulated product administration by proposed routes to one or a few mice prior to undertaking full efficacy experiments. The adequateness of degrees of phage purification, however, should be assessed with increasing rigor the further along in its development that a formulated product has been brought, e.g., in vitro testing versus animal testing involving few animals, versus animal testing involving more or different types of animals, versus clinical testing.

#### 2.3.3. Dilution

Were formulations diluted prior to storage? Were formulations further diluted prior to use? In either case, diluted into what and in what proportions? If phages were concentrated at some point then the details of concentration steps should be reported as well, including in terms of degrees of phage losses observed. Note that dilution can be viewed not just as a means of reducing phage concentrations but also as a means of increasing formulated product purity. Thus, the level of purity of formulated products can vary with degree of phage dilution, as for example lysis products are reduced in concentration, and this issue can be especially relevant the less pure phages are prior to dilution. Reasons for diluting phages prior to administration should also be indicated. For example, were there safety or efficacy concerns associated with administering too many phages? Was there perceived utility in applying phages within a larger total volume such as in terms of a lavage? Were phages diluted into an animal’s food or water supply? Were phages diluted in anticipation of future or even present economizing in terms of total phage numbers used per administration? Keep in mind in any case that the extent to which phages are diluted, overall, should be fully under the experimenter’s control especially since phage concentration steps can precede phage dilution steps. Phages also will tend to be diluted in the course of delivery (i.e., dosing) and it can be important to take into account dilution-association reductions in phage densities as an aspect of phage therapy pharmacokinetics.

#### 2.3.4. Ingredients

What ingredients were mixed with phages to generate formulated products? What were the purposes of the individual ingredients used? For example, what ingredients served as pH buffers, virion stabilizers, osmotic balancers, preservatives, etc.? The usefulness of explicitly indicating this information is not only for the sake of conveying knowledge but also so that authors themselves may spend time pondering the utility of different ingredients, as well as comparing their appreciation of the importance of specific ingredients with how others have considered these utilities. The non-phage ingredients of formulated products, in other words, can be relevant to product performance and therefore should merit at least some consideration versus time devoted solely to understanding phage properties. Furthermore, formulation ingredients may impact different phages in different ways, or different phage uses in different ways, and therefore may be important to consider towards the optimization and/or debugging of phage therapy protocols.

#### 2.3.5. Physical State

Were formulations used in the form of liquid suspensions or instead in a solid phase such as delivered as tablets or instead as dry powders? Was the intention of employing phages in the state used one of improved delivery, improved storage, economizing, or some combination or additional criteria? In comparing products formulated to different states, were differences in phage titers observed at relevant sites following delivery? In other words, even given delivery of similar numbers of phages as determined in vitro, it is possible that the equivalence of those numbers may not hold as a function of physical state during application, that is, following delivery to, for example, an animal. What is, therefore, the outcome of actual dosing in terms of in situ phage titers especially as ultimately found in the vicinity of targeted bacteria?

#### 2.3.6. Processing

If formulations were generated by more complex methods than simply mixing ingredients, then what specifically were the processes involved? Even with simple mixing of ingredients, if end-users are responsible for this processing, then to what degree is the precision of such processing controllable or might phage performance be impacted by variation in procedures? For example, if a formulated product must be thawed or dissolved prior to use, then the temperature or rapidity of these processes could vary, potentially affecting the resulting phage titer. Minimally, if any such processing occurs, even during preclinical testing, then it should be done in a consistent as well as well-described manner.

#### 2.3.7. Disclosure

If any of the above represent especially unpatented proprietary information, and therefore is not provided within a publication, then this should be explicitly stated. Alternatively, patents or pending patents should be appropriately referenced. Information necessary to repeat experiments should not, in other words, simply be left out of manuscripts without indication or explanation.

### 2.4. Titers

Pharmacologically, it is crucial that readers know explicitly how much of each ingredient is present within formulated products, and no ingredient is more relevant to phage therapy success than phages themselves. The concentration of phages in formulated products should be described in terms of titers, that is, numbers of phages present per mL or, for phages formulated into solid products, then per gram.

#### 2.4.1. Assessment

As phages in phage therapy are being applied as drug equivalents, phage concentrations ultimately should be presented as within-product titers (that is, concentrations). Thus, how many phages—as measured either in plaque-forming units (i.e., PFUs), or instead as killing titers for inviable agents (such as non-lytic engineered phages)—were unambiguously present per unit volume, or per gram if delivered in a dried state, in formulated products at the point of dosing? How, specifically, were these titers determined? Useful information can include the indicator host strain, the media employed including in terms of indicator bacteria propagation, temperatures, the physiological state of hosts at the point of mixing with phage (e.g., log phase versus overnight cultures), how and for how long indicator bacteria were stored prior to use, and, indeed, the various volumes involved, etc. It can be helpful as well to indicate titers for phage types found within crude or purified stocks as used to generate formulated products. For example, “phages were diluted into individual, single-phage stocks of 10^9^ PFU/mL prior to generation of finalized products.” For online calculators for computing phage titers as well as killing titers, see [17,18].

#### 2.4.2. Within-Formulated Product Information

Indication of within-formulated product titers can be particularly useful when products consist of multiple phage types, i.e., when formulated as cocktails. This is rather than solely indicating amounts added towards the generation of formulated products. For example, stating “stocks of three phages were combined to generate a cocktail of 10^9^ PFU/mL” or “three phage stocks of 10^9^ PFU/mL were combined” does not always supply to readers, or at least this reader, sufficient information to fully appreciate within-formulation titers. Preferable would be to unambiguously indicate, for example, that 3.3 × 10^8^ PFU/mL for each of the three phages added were present after mixing, for a total of 1 × 10^9^ PFU/mL, rather than a total of 3 × 10^9^ PFU/mL or instead different titers for different phages. This exercise is important especially since not all phages within a cocktail may be active against a given target bacterium. Thus, a cocktail consisting of ten phage types, only one of which is active against a specific bacterial strain, may have an active titer that is only one-tenth the titer of the cocktail as a whole (so, ideally, the reader will unambiguously know what this titer is).

#### 2.4.3. Stability

Of particular importance is the extent to which phage titers decline over time prior to application, and also whether titer declines are accompanied by losses of phage bactericidal activity (the latter, i.e., would be declines in plaque-based titers versus declines in killing titers). Thus, were titers determined over the course of storage and/or were titers observed to change? What were the details of phage storage including temperature, access of light, humidity (the latter especially if phages are stored within unsealed containers but also if phages are stored in a dried/solid state), properties of storage containers, etc., and how long were formulated products stored prior to use? Was this duration varied between experiments? If so, then how was it varied, and why was it varied? In addition, how soon prior to experimentation were titers last determined? If formulated products consist of phage cocktails, then were differences observed in titer stability among phages during this storage? As development of a phage product progresses, then the question of how transportation or user-associated variation in storage conditions can affect phage stability also can become important. Note that the stability of the non-phage ingredients of formulated products could be relevant as well.

### 2.5. Subjects

Treatment subjects are the organisms, such as animals, human patients, etc., which are subject to phage therapy treatment. In addition, with phage-mediated biocontrol, instead environments can be the subjects which are treated, or foods, and consideration of these may be included under this heading as well.

#### 2.5.1. Specifics

What are the basic details of treatment subjects including species, breed, age, gender, any pharmacogenetic information, and weight? Weight especially is for the sake of dosing uniformity—particularly following other than local phage application to a wound—and is important towards easing the translation from preclinical to clinical experimentation. Also important is a description of how subjects were housed and cared for, or, in the case of biological control, the specifics of environments to which phages were added, including temperature, humidity, and also degree of exposure to sunlight. In addition, by what criteria were individuals (especially for clinical treatment), types of test subjects, or conditions chosen?

#### 2.5.2. Health

What was the health status of subjects prior to the bacterial challenge? Alternatively, if naturally-occurring infections were treated, then for how long were infections known and what prior treatments of subjects were undertaken? Especially for clinical therapy, this includes what drugs such as antibiotics individuals were being treated with, or aspects of lifestyles which might affect health. Indeed, it is of course important in any case to match controls with experimental groups in terms of these various criteria.

#### 2.5.3. Numbers

How many subjects were included in the study? Numbers of subjects are important towards achieving statistical experimental validity. This includes numbers of control as well as numbers of experimental subjects. Note that controls can include not just untreated controls but also unchallenged controls, the latter potentially consisting of both phage- and not-phage-treated subjects. By contrast, it is important as well to not allow experiments to become so large that they are no longer manageable or economically feasible. Consultation with a statistician over the course of the design of experiments can be helpful, as too can simply thinking through the utility of various controls or experimental groups. Doing experiments in stages also can be useful, if possible. This potentially can allow for debugging of experiments prior to their scaling up to full size, e.g., as suggested above for preliminary toxicity testing of formulated products. Explanation of the thinking underlying what numbers were used at what stages, even if only in brief, can be helpful to fellow researchers as well.

### 2.6. Bacteria

What are the details of the bacteria used in the laboratory, of bacteria in the course of infection, and of bacteria upon phage treatment? 

#### 2.6.1. Strains

What bacterial strain or strains were used? If so designated, then provide strain names, sub-types, nucleotide database and/or culture collection accession numbers, relevant descriptive publications, etc. Antibiotic resistance characteristics can also be important. Some documentation of a bacterium’s relevant past use may be helpful including as phage hosts, in disease models, or as clinical isolates. For bacteria isolated specifically for a phage study, then how were they isolated, from what sources, how were they characterized including taxonomically, and why were these specific bacteria chosen? Alternatively, from whom were the bacteria obtained? Note also the utility of employing the bacterial strains along with the experimental details used in equivalent disease models by other authors. That is, even if these other studies did not involve phage therapy and even if the bacteria employed, though the same species, were not those against which to-be-tested formulated products were specifically developed, it is important towards phage therapy development to achieve some continuity with antibacterial development more generally. Some demonstration of the breadth of bacterial strains within a given species, against which a formulated product can achieve positive results during actual phage therapy experiments, is important as well. 

#### 2.6.2. Preparation and Physiology

How were bacteria prepared prior to the bacterial challenge and why was this method chosen? For example, with shaking (at what speed?), or as grown on solid media. What temperature? Employing what media? For how long, etc.? Were cultures subsequently refrigerated or otherwise manipulated prior to use? What was the resulting physiological state of the bacteria upon challenge, e.g., log phase or stationary phase? If stationary phase, then for how long into stationary phase were bacteria incubated prior to application? Note that simply stating “overnight cultures were used” lacks precision. Indeed, it can be helpful for the sake of the reproducibility of experiments to supply a detailed description of precisely how bacteria were prepared, and this is especially so since even such terms as log phase and stationary phase can lack precision and thus replicability. In addition, for many bacteria, oxygen is a limiting factor. If so, then different flasks along with speed of shaking can result in variation between experiments. If oxygen concentrations during bacteria preparation were measured, then that too should be reported. Alternatively, robustness of results to variation in conditions, such as variation in the preparation of challenge bacteria, can be valuable, though only to the extent that adequate record keeping and reporting of known variation is practiced.

#### 2.6.3. Concentrations

Care should be taken in the use of concentration units for bacteria since numbers of colony-forming units can mean different things for different bacteria under different conditions, e.g., broth culture *Escherichia coli* versus *Staphylococcus aureus* grown on solid media. Notwithstanding that caveat, however, one should provide a detailed description of how bacterial concentrations were assayed so that others can have the option of repeating these processes in precisely the same way. More generally, for the sake of repeatability, it can be important to make sure that measurement precision is good, and repeatable, even if what exactly constitutes a colony-forming unit is not always accurately appreciated. Accuracy on a per-cell basis, however, can be important for quantitatively appreciating phage-bacterium interactions. For the latter, see [19].

#### 2.6.4. Challenge

What was the route of bacterial challenge? What were the specifics of how the bacterial challenge was delivered? What were the numbers and volumes applied? If known, then what is the infectious and/or lethal dose for the bacterial strain and challenge route used, how were these numbers determined or confirmed for the current study, how did the actual challenge compare with these numbers, and what were the criteria if any for dosing as relative to these numbers? For example, for the latter this could be precedence in other phage therapy or antibiotic treatment studies. Note that a good description of the normal course of infection following bacterial challenge, i.e., as used in the reported experiments, can serve as a substitute for determination of infectious or lethal doses. Indeed, such descriptions can be valuable even given infectious- or lethal-dose determinations, and this is true as well even if good justification cannot be provided for why a specific extent or route of bacterial challenge was used. Alternatively, if preexisting, that is, naturally occurring infections were treated, then details of infection pathophysiology and microbiology should be provided. In any case, what was the state of the bacterial infection at the point of initiation of phage treatment, e.g., presence of biofilms, display of antibiotic tolerance, and/or bacterial densities?

#### 2.6.5. Enumeration

How were infections characterized microbiologically? Was an effort made to determine whether enumerated bacteria were still susceptible to treatment phages? That is, might phage treatment have resulted in selection for phage-resistant bacteria? Especially if still phage-sensitive, then were efforts made to protect bacteria from phages in the course of bacterial enumeration? The issue with this latter point is that bacterial enumeration typically involves destruction of the “spatial structure” of infections, which can allow phages to reach and kill bacteria that may not otherwise have been reached by phages prior to enumeration. The result can be false determinations (overestimations) of degrees of bacterial killing in the course of in situ phage treatment. To avoid this outcome, one can rapidly dilute bacteria upon sampling and also employ specific anti-phage ingredients such as anti-phage serum or virucidal but not bactericidal agents within diluent. In addition, mock target bacteria may be used as bacteria-survival enumeration controls. In any case, it is important to be conscious of needing to protect bacteria from phages during bacterial enumeration, and possibly also protection of phages from bacteria during phage enumeration.

### 2.7. Dosing

Dosing refers to the details of separate applications of phage-containing formulated product to subjects.

#### 2.7.1. Initiation

What was the timing of the bacterial challenge, or detection of the preexisting infection, relative to the initiation of phage dosing? Generally, phage application prior to bacterial challenge is considered to be prophylactic. Phage application simultaneous with bacterial challenge either can be viewed as an optimized proof of principle, if phages are applied to the same location as bacteria, or instead can serve as a means of testing, again in an optimized manner, of the potential for phages applied in one location to reach bacteria applied to another (the latter thus being a pharmacokinetic analysis). If a delay exists between bacterial challenge and the initiation of phage dosing, then this can be viewed as an effort to mimic phage treatment of existing disease. It is incumbent upon authors, however, to provide evidence that indeed the desired disease state, e.g., a chronic bacterial infection, in fact was present at the point of initiation phage application. In particular, it can be useful to distinguish bacterial contamination or colonization from the establishment of clinically relevant infection in terms of the state of disease at the point of initiation of phage dosing.

#### 2.7.2. Numbers

What was the volume of the phage formulated product applied and thereby, with titer unambiguously stated (as discussed above), the total number (i.e., dose) of phages of each type? Why was this dose selected? Note that generally in phage therapy there are three key variables: state of infections at the point of phage application (ideally serving as a controlled variable especially given initiation of infections via bacterial challenge), consequences of phage application (the dependent variable such as efficacy or toxicity), and numbers of phages applied (the independent variable), the latter ideally described in terms of both volumes and titers (or grams and phage concentrations for dosing with phages applied in solid phase). Generally in publications one sees consequences most consistently reported, the precise state of infections at the point of phage application not necessarily reported, and, in a number of studies, actual numbers of phages applied per dose sometimes ambiguously presented. This latter point I return to below.

#### 2.7.3. Administration

What was the route or routes of dose administration? For example, topical application directly to a bacterial infection, delivery via injection around an infection, aerosolized delivery to the lungs (which also is a form of topical delivery), via ingestion to the gastrointestinal tract, per os (that is, ingestion with the goal of systemic delivery), parenteral application, etc. Include discussion of alternative routes that might be employed, as well as, possibly, why they were not used. Also important are routes of administration normally used clinically for application of standard antibacterials (i.e., antibiotics) as well as what may have been used clinically for phages. The latter may be gleaned from older, e.g., 1930s and 1940s phage therapy literature (e.g., see [20]). How too was phage dosing accomplished in terms of methods and technology? 

#### 2.7.4. Densities In Situ

Though not necessarily in fine-grained terms, one nevertheless should be at least conscious of the utility of knowing what levels of phages are present in the vicinity of target bacteria following dosing. Thus, what, if it is possible to determine, were those levels? Data such as these serve as important measures of phage therapy pharmacokinetics (below). In addition, what efforts if any were made to improve upon, that is, to increase post-application phage densities as found in the vicinity of target bacteria? For example, debridement may have been employed or alternatively different application routes may have been attempted toward optimization of pharmacokinetics. Exploring how to increase phage densities in the vicinity of target bacteria can be important even if based only on theory or judgement, though underlying reasoning should be specified and, as appropriate, cited. Improvements also may be attempted even if the measuring of resulting phage densities in situ is not easily accomplished, e.g., with efficacy improvements serving as surrogate indicators. Such efforts towards pharmacokinetic improvement of phage therapy protocols should be reported and their potential to quantitatively improve phage penetration to target bacteria discussed.

#### 2.7.5. Multiplicity of Infection

The definition of multiplicity of infection (MOI), if MOI predictions are used in studies, should be explicitly stated. Historically this is the number of phages which have been shown to have *adsorbed* bacteria divided by the number of target bacteria. Alternatively, but historically inaccurate, this could be the number of phages only *added* to bacteria, again as divided by the number of target bacteria. The actual definition must be indicated because these contrasting perspectives can provide enormously different values depending on rates of phage adsorption to bacteria in combination with the time frames over which adsorption has been allowed to occur. MOI, again *if* this value is calculated, also should be rigorously defined in terms of numbers of target bacteria. Is this the number present at the point of phage application, for example, or instead the number of bacteria present at the point of bacterial challenge? For prophylactic phage treatment, then similar questions can be asked, that is, is this the number of phages present at the point of phage application or instead the number of phages present at the point of bacterial challenge? Furthermore, there often is no way of knowing whether all target bacteria are equally available in situ to provided phages, though nevertheless equivalent adsorbability is an implicit assumption of MOI-based predictions.

#### 2.7.6. Prediction

The utility of using MOIs as a measure of phage dosing is based on associated statistical inferences, specifically prediction of the fraction of bacteria which are expected to remain unadsorbed by added phages. This fraction is equal to the expression, e^−MOI^, with MOI as traditionally defined and assuming that phages adsorb Poissonally. Given that such predictions are the key utility of using MOI-based characterization of phage dosing, this calculation should therefore be reported in conjunction with MOIs. For example, an MOI of five adsorbed phages per targeted bacterium should correspond to survival of slightly less than 1% of these bacteria (=e^−5^). In the absence of such reporting then either the reader is forced to do the calculations themselves, and not always with sufficient information provided, or instead the naïve reader might reach false conclusions as to the adequacy of MOIs used. In any case, MOI-based predictions are useful only to the extent that (1) MOIs have been determined in an historical sense (i.e., as based on number of phages which have actually adsorbed bacteria); (2) that substantial phage in situ phage replication is not expected (since otherwise it may be impossible to appreciate actual MOIs achieved during treatments); and/or (3) if there is an interest in determining whether the phage impact on bacteria is greater (or worse) than that expected based on the MOI achieved, or at least the MOI assumed. See [21] for further guidance as to the implementation of these statistics.

#### 2.7.7. Report Titers!

As considered above, phage titers during dosing along with what phage volumes, or masses, were applied during dosing should *always* be reported, without exception, in phage therapy studies. In pharmacology the most important treatment-associated information, besides subject characteristics, generally is drug description (what is being applied) and dose (how much is applied as well as how). MOI-based dosing, however, does not provide the latter’s quantitative information in a sufficiently straightforward manner. MOI, from a pharmacological perspective, consequently is never an acceptable *sole* measure of dosing. Therefore, always report phage dosing in terms of volume (or grams) along with titers (or concentrations) of phages applied*.* By way of illustration of these points, imagine the uproar were descriptions of antibiotic dosing limited to ratios of active units to ambiguously or imprecisely described numbers of bacteria, or the difficulties in translating such descriptions to real-world use where bacterial densities at the time of antibacterial administration often are even less-well appreciated.

### 2.8. Treatment

Treatment is the collective manner of dosing, particularly as consisting of single or instead multiple phage doses and, for the latter, at what frequency, etc. Treatments also can involve more than just dosing with phage formulated products. The initiation of the phage application component of treatments is covered under dosing.

#### 2.8.1. Preparation

How were subjects prepared for treatment? For example, stomach acid reduction or tissue debridement could have been used. What was the length of the interval between preparation and phage dosing? Indeed, did preparation take place prior to each dosing, assuming multiple dosing, or instead only prior to the first dosing?

#### 2.8.2. Numbers of Doses

Treatments may consist of a total of only a single dose versus two doses versus more than two doses, or instead can consist of continuous application (the latter consisting of ongoing fluid application along with drainage). If not continuous dosing, then what was the total number of doses applied over the course of treatment? Were the number of doses applied predetermined? What criteria, predetermined or otherwise, were used to decide on this number or approach? Was there any variation between doses, e.g., such as differences in volumes applied or alterations in delays between? If only a single dose was applied, then what was the justification for not applying additional doses?

#### 2.8.3. Frequency

If more than one phage dose was applied, then how often were phages applied? What criteria were used to justify this frequency? If the frequency was continuous, then by what means was ongoing phage application achieved? Intermediate between multiple dosing and continuous is periodic phage application but in a manner in which phage release is not instantaneous. In this case, how often if at all were these materials changed or reapplied? At more sophisticated levels of characterization, one could also consider the rate of phage release, rate of depletion from these materials, or, more generally, rates of phage decline in situ following dosing (though the latter can be complicated for phages versus conventional drugs since phage increases in density, in situ due to phage replication, is possible as well).

#### 2.8.4. Duration

What was the total length of time over which treatments were continued? For example, multiple days or multiple weeks? What criteria were used to justify this duration? Was this length of time predetermined, or was this instead based, for example, on observed rates of improvements in infections? Even if treatments consisted of only a single phage dose, the duration over which monitoring takes place should be reported, along with justification of this duration and whether or not it was predetermined.

#### 2.8.5. Other

What non-phage materials or methods were employed in the course of treatments? What was the reason for their use? What impact may they have had on phage-associated activity and/or treatment success? In addition, were controls performed in which these non-phage materials were not used, or alternatives used, so as to explicitly address the contribution of these materials to observed efficacy as distinct from phage presence? An example of a non-phage material would be use of antibiotics or analgesics, though in fact so can various otherwise standards of care serve as non-phage aspects of treatments such as the application of bandages, etc.

### 2.9. Pharmacology

Pharmacology can be differentiated into pharmacokinetics (body impact on drugs) versus pharmacodynamics (drug impact on body). Pharmacokinetics involves exploration of changes in drug concentration throughout the body as a function of time, particularly at the site(s) of drug targets, and to a degree this issue has already been considered here for phages during phage therapy. Pharmacodynamics includes both efficacy considerations (see next section) and negative effects of drug application, i.e., side effects and toxicities.

#### 2.9.1. Pharmacokinetics

Were changes in phage densities in various body locations monitored, particularly given systemic phage application? Did phage densities increase, decrease, or instead stay the same especially in direct association with bacterial infections over the course of treatments? How did changes, if monitored and found to be present, coincide with dosing timing or treatment efficacy? Were phage in situ concentrations—especially as seen without presence of target bacteria—potentially adequate for passive treatment, that is, bacterial eradication without relying on in situ phage replication to higher numbers? For one means of addressing the latter, see, for example, [21]. Note that though detailed pharmacokinetic analyses are not essential for all studies, certainly speaking to pharmacokinetics to some degree can be useful, or otherwise thinking about the potential effectiveness of different approaches to delivering sufficient phage titers to the locations of target bacteria.

#### 2.9.2. Toxicities

What toxicities were observed and in what frequencies? Side effects may be associated with phage application to unchallenged controls or as seen during the course of treatment, and these context distinctions should be explicitly reported. Were toxicities observed when employing phage-free, i.e., mock-formulated products? Alternatively, were toxicities observed with formulations from which phage virions had been removed, thus, ideally, retaining any lysis products which may have been present in the original formulation? If using cocktails, were toxicities associated with any one particular phage type or stock, e.g., with the latter, when different bacterial hosts were used for the propagation of different phages making up the cocktail? If toxicities appear to be formulation- rather than phage-associated, then could the responsible ingredients be isolated? Did degrees of toxicities differ from those seen with alternative treatments, e.g., such as while employing antibiotics, or using different routes of phage application? Did toxicities vary with degree of efficacy observed? Also, if subjects died, then what were the observed causes of their mortality? In general, in other words, it is important to address whether any toxicities, side effects, or mortality, if observed, may be distinguished from negative aspects of the bacterial infection and its treatment, as well as from the impact of non-phage formulation ingredients, rather than necessarily explicitly being a consequence of using phages as antibacterial agents.

### 2.10. Efficacy

Were phage treatments successful and how was success defined as well as monitored?

#### 2.10.1. Results

What were the efficacy results of phage treatment? To what degree and under what experimental circumstances may efficacy have varied? How was efficacy determined, e.g., such as in terms of improvement in health or instead solely in terms of reductions in bacterial numbers? If by health, then by what criteria was degree of health determined? To what extent did this efficacy coincide with microbiological improvement? Importantly, in some cases symptoms associated with infections may be relieved without completely eliminating targeted bacteria. If symptomatic improvement occurs without substantial microbiological improvement, then it is possible that something other than phage bactericidal effects were responsible for that improvement or, alternatively, that distinctions exist between the bacterial populations being monitored, i.e., phage-sensitive bacteria prior to treatment versus phage-resistant bacteria during or following treatment.

#### 2.10.2. Comparison

Comparison minimally should be between phage-treated and not- or placebo-treated groups and statistical comparisons of course should be made. In addition, it is permissible to include comparisons between different types of phage treatment—different phages, different formulations, formulated products but with various individual non-phage ingredients excluded, different dosing routes, etc.—and indeed different types of phage-free controls. Also of importance, however, is the question of whether observed results, if indeed positive, would be considered to be adequate under real-world circumstances, i.e., biologically or clinically significant outcomes rather than solely statistically significant ones. Furthermore, how may have observed efficacy compared with standard of care, i.e., established intervention, including relative to antibiotic application as an additional control? Thus, and importantly, there not only are variations in terms of what statistically significantly positive results may involve, i.e., as can vary in terms of comparison groups, but also it is a very relevant question whether results, even if statistically significant, may be clinically meaningfully extrapolated. 

#### 2.10.3. Activity

Based especially on the use of proper controls, can observed efficacy be explicitly attributed to phage presence in formulations? Furthermore, can phage action be unambiguously attributed to phage infection of bacteria in the course of treatment? For the latter, for example, were increases observed in phage numbers in the vicinity of target bacteria above those accounted for by dosing, i.e., as indicating phage replication? Alternatively, was bacterial lysis and/or substantial increases in phage-resistant bacteria observed? In addition, were levels of bacteria killing in line with, greater than, or less than as expected based on killing titer calculations? See [18,21] for the latter.

### 2.11. Discussions

Discussions consider derived aspects of results as well as how results either were improved in the course of a study or might be improved upon in the future. In addition, how do experiments or results compare with those reported by others? The phage therapy world is a relatively small one so there should be little reason not to at least mention and ideally to compare results with those obtained using similar bacteria, infection models, or treatment approaches. See, for example, [20] for an extensive list of phage therapy publications.

#### 2.11.1. Comparisons

In what way was the experimental design reflected (or contrasted) in the efforts of others? In what way were results reflected (or contrasted) also in the efforts of others? How did the results obtained compare with those observed using more standard treatments, that is, such as antibiotic treatment? What was the impact of using different numbers, such as different titers of dosed phages, if indeed such data were obtained? Similarly, what was the impact of using different phages, if so considered, and perhaps particularly in light of differences in phage in vitro properties? What was the impact of using different numbers of doses per treatment, different dosing intervals, different routes of dosing, or other treatment differences? Consider what may have been the underlying bases of any differences in outcomes observed.

#### 2.11.2. Limitations

What may be the limitations of using different approaches and/or on what bases were different approaches *not* used? For example, in certain animal models it can be difficult to treat using multiple phage doses. Alternatively, how might these limitations be overcome? Indeed, what potential may there be for improvement of results by employing different phage therapy approaches? Lastly, in terms of limitations is the question of validity, an issue which here has been touched upon though not extensively addressed. That is, it is always important to actively question the potential for a given experimental system to effectively mimic whatever it is that it is intended to model.

## 3. Conclusions

It is unlikely that the criteria proffered here are exhaustive. Nonetheless, and ideally, they may serve as guideposts towards more effective reporting of phage therapy experimentation. In particular, currently there are no accepted, official standards for phage therapy experiments. While not seeking to define such standards—as that preferably would involve input from multiple experts—it is hoped that the suggestions presented will help researchers in their thinking during experimental design, analysis, and presentation of results. Ideally the outcome will be a more useful phage therapy literature, more effective experimental design, greater impact of individual studies on the development of the field, increased translation of experimental efforts into clinical success, and/or simply the supplying of sufficient information to allow for precise repeating as well as extension of published studies.
